# Compensation Preferences: The Role of Personality and Values

**DOI:** 10.3389/fpsyg.2021.550919

**Published:** 2021-04-07

**Authors:** Amanda M. Julian, Onno Wijngaard, Reinout E. de Vries

**Affiliations:** ^1^Department of Psychology, University of Calgary, Calgary, AB, Canada; ^2^Heineken International B.V., Amsterdam, Netherlands; ^3^Department of Experimental and Applied Psychology, Vrije Universiteit Amsterdam, Amsterdam, Netherlands

**Keywords:** HEXACO personality, values, compensation, compensation preferences, personality

## Abstract

The present study investigated relations between personality and values on the one hand and compensation preferences on the other. We hypothesized that HEXACO Honesty-Humility and self-transcendence *versus* self-enhancement values predict preference for higher relative compensation level and that HEXACO Openness to Experience and openness to change *versus* conservation values predict preference for compensation variability. Furthermore, we expected perceived utility of money and risk aversion to mediate the respective relations. The hypotheses were tested using a sample of 2,210 employees from a large international organization. The results provided support for the direct and mediated relations between personality and values on the one hand and preferences for compensation variability and level on the other.

## Introduction

In recent years, the world has witnessed numerous instances of public outrage over high corporate salary levels and bonuses, leading to a spirited debate of the societal consequences of such remuneration practices. Gaining insight into potential drivers of the level and variability of employee salaries may be important not only for a company’s reputation but also for its recruitment, selection, and career advancement practices. To shed light on the forces contributing to the demand for these aspects of compensation, the present study examines dispositional drivers of employee compensation preferences. More specifically, we explore the role of individual personality traits and values in determining employee preferences for both higher relative compensation level [*i*.*e*., compensation level compared to others (coworkers/others in a similar role)] and compensation variability. Furthermore, mediating mechanisms are examined to gain a more nuanced understanding of the relations.

### Personality

We propose that two HEXACO personality traits, Honesty-Humility and Openness to Experience, are especially relevant in predicting preference for higher relative compensation level and variability, respectively. Whereas additional personality traits belonging to the HEXACO framework may also be relevant to compensation preferences, we chose to focus on those deemed most important in the prediction of the criteria of interest in this study. Honesty-Humility is defined by “honesty, fairness, sincerity, modesty, and a lack of greed” for those who score high on the trait (Lee and Ashton, 2004; p. 332). In other words, high scorers on the trait are unwilling to try and manipulate others, unlikely to cheat or lie, are modest and unassuming, and are uninterested in lavish wealth or the possession of luxury goods. On the other hand, Honesty-Humility has been found to be negatively related to materialism (Ashton and Lee, 2008) and narcissism (Lee and Ashton, 2005), and therefore low scorers are more likely to assign importance to material goods/possessions, to feel superior to those around them, and to feel entitled to better treatment and privileges that are not afforded to others, compared to those scoring high on the trait. As low scorers on Honesty-Humility are more likely to be greedy, selfish, and entitled, it is expected that these individuals will prefer higher levels of compensation compared to others (*i*.*e*., others in their organization and in their specific job).

Hypothesis 1. Honesty-Humility is negatively related to preference for higher relative compensation level.

Openness to Experience is defined by aesthetic appreciation, inquisitiveness, creativity, and unconventionality (Lee and Ashton, 2004). In other words, high scorers on this trait have an appreciation for the beauty in art and nature, have a tendency to seek out new information and knowledge, prefer innovation and originality to the *status quo*, and are open to unusual or radical ideas. According to Ashton and Lee (2007), one of the likely benefits of high levels of Openness to Experience is the obtainment of material and social gains through discovery.

As increasingly variable compensation structures introduce higher levels of risk and uncertainty, it is unsurprising that previous research has linked risk aversion to preference for fixed *versus* variable compensation schemes (Cable and Judge, 1994). Openness to Experience has been consistently linked to risk-related variables in previous research. For instance, individuals scoring high on Openness to Experience have been found to be more likely to engage in sensation seeking and risk-taking behavior (De Vries et al., 2009), to tolerate risk in order to achieve a gain in an experimental task (Lauriola and Levin, 2001), and to perceive benefits of/participate in social and recreational risk-taking (Weller and Tikir, 2011). Indeed previous research has demonstrated that five-factor Openness to Experience (nearly identical to HEXACO Openness to Experience) positively predicts employes’ attraction to variable compensation schemes (Vandenberghe et al., 2008). Consequently, a similar relation is expected in the present study.

Hypothesis 2. Openness to Experience is positively related to preference for compensation variability preference.

### Values

Whereas personality traits refer to consistent patterns of thoughts, emotions and behaviors (McCrae and Costa, 1990), values refer to persisting goals depicting what an individual deems important (Roccas et al., 2002). Schwartz (1992, 1994) advanced a circumplex of human values containing two bipolar value dimensions, which include self-enhancement *versus* self-transcendence and openness to change *versus* conservation. The two-value dimensions are conceptually similar to, though by no means indistinguishable from, HEXACO Honesty-Humility and Openness to Experience, respectively (Lee et al., 2009).

The self-enhancement *versus* self-transcendence axis contrasts values reflecting a pursuit of success, status, and dominance of both people and resources (values of power, achievement, and hedonism; Schwartz, 1994) to those emphasizing acceptance and concern for others (values of universalism and benevolence), respectively. It is anticipated that individuals endorsing values within the self-enhancement pole will prefer higher compensation, given their tendency to be highly oriented toward personal success and achievement (Schwartz, 1994), and one indicator of success within an organization is one’s level of compensation compared to others. Additionally, individuals endorsing a power motive are likely to exhibit a desire for recognition and prestigious possessions (Schwartz, 1994; Winter, 1973), which may be attained through higher levels of compensation relative to others.

Hypothesis 3. Self-transcendence (*versus* self-enhancement)^1^ is negatively (positively) related to preference for higher relative compensation level.

The openness to change *versus* conservation axis contrasts values reflecting a preference for independence and novelty (values of self-direction, stimulation, and hedonism; Schwartz, 1994) to those emphasizing stability, self-restriction, and maintaining the *status quo* (values of security, conformity, and tradition), respectively. As mentioned previously, individuals who are drawn to novelty and new experiences are often more eager to take risks (De Vries et al., 2009). Moreover, previous research has demonstrated a link between stimulation and hedonism (which shares elements with both openness to change and self-enhancement dimensions) values and engagement in risk-taking behaviors (Athota et al., 2017; Dollinger and Kobayashi, 2003). Given that highly variable compensation systems present increased levels of risk with reward, individuals endorsing values subsumed by openness to change will likely prefer more variable compensation schemes. Those endorsing values within conservation, however, would be expected to be less tolerant of the increased uncertainty associated with more variable compensation (Cable and Judge, 1994).

Hypothesis 4. Openness to change (*versus* conservation) is positively (negatively) related to preference for compensation variability.

### Mediating Mechanisms

#### Perceived Utility of Money

Whereas compensation may comprise non-monetary components, oftentimes it predominantly consists of financial-based rewards (Milkovich et al., 2010). Therefore, the degree to which money is perceived as important or valuable is likely to relate to individuals’ preference for higher relative compensation level. Moreover, a strong inclination to prioritize money is also likely to characterize individuals who score low on Honesty-Humility and those who endorse self-enhancement values.

Honesty-Humility has demonstrated negative associations with costly displays of wealth (Lee et al., 2013), engagement in risky behaviors to achieve wealth (Ashton et al., 2010), and materialism (Ashton and Lee, 2008). Previous research has also demonstrated a negative relation between five-factor Agreeableness, which contains some Honesty-Humility content (Ashton and Lee, 2005), and the consideration of money as important (Matz and Gladstone, 2018). Individuals who endorse values subsumed by self-enhancement are also expected to prioritize money as valuable, as increases in capital would be expected to satiate the desire for personal gain and power (Schwartz, 1994). Consequently, it is anticipated that the relations among Honesty-Humility and self-transcendence (*versus* self-enhancement) and preference for higher relative compensation level will be explained by individuals’ perceived utility of money.

Hypothesis 5. Perceived utility of money mediates the negative relations between (a) Honesty-Humility and preference for higher relative compensation level and (b) self-transcendence (*versus* self-enhancement) and preference for higher relative compensation level.

#### Risk Aversion

Previous research has highlighted risk aversion as a predictor of preference for fixed *versus* variable compensation schemes (Cable and Judge, 1994). Additionally, risk aversion seems to moderate relations among “control-by-pay” performance-based systems and employee withdrawal cognitions, pay satisfaction, and organizational citizenship behaviors (Deckop et al., 2004). Furthermore, risk attitudes appear to moderate the relation between pay-mix and reward valence (Pappas and Flaherty, 2006). Thus, it appears that individuals’ inclination toward risk is likely to impact one’s receptivity to compensation variability.

As mentioned above, previous research has demonstrated a positive association between Openness to Experience and risk-related variables (*e*.*g*., De Vries et al., 2009) as well as value content within openness to change and engagement in risky behaviors (Dollinger and Kobayashi, 2003; Athota et al., 2017). Therefore, whereas both dispositional variables are linked to some affinity toward (or avoidance of) risk and whereas risk-related attitudes have been linked to a preference for variable compensation, research has yet to examine such a mediated relation.

Hypothesis 6. Risk aversion mediates the positive relations between (a) Openness to Experience and preference for compensation variability and (b) openness to change (*versus* conservation) and preference for compensation variability.

## Materials and Methods

### Sample and Procedure

The sample consisted of employees of a large multinational fast-moving consumer goods company. All 3,638 employees working in 12 operating companies across 12 countries were invited to participate in the study *via* email invitation.^2^ Of those, 2,210^3^ provided complete questionnaires and were retained. The respondents were 23.7% female (524), the mean age was 41.15 years (SD = 8.30), and the mean organizational tenure was 12.42 years (SD = 8.64) (For additional demographic information, see Supplementary Table A1). Data collection took place between February and June of 2012. Participation was not completely anonymous, as the participants were asked to provide their names to the researcher conducting the data collection. However, they were assured that individual-level data would be treated confidentially.

### Measures

Because of company restrictions to the length of the questionnaire, only items of the hypothesized constructs were included in the survey. English questionnaires were translated into Dutch, French, Greek, Italian, Polish, Brazilian, Portuguese, Romanian, Russian, and Spanish using a translation–back-translation procedure. When available, existing translations of the Honesty-Humility and Openness to Experience scales were used. The responses were indicated on a five-point scale ranging from 1 (strongly disagree) to 5 (strongly agree), unless otherwise indicated. The reliabilities are presented in Table 1.

**TABLE 1:** Means, standard deviations, intraclass coefficients and zero-order correlations (raw data).

	*M*	*SD*	Range	ICC(1)	1	2	3	4	5	6	7	8	9	10	11	12
1. Age	41.15	8.30	23.00-66.00	–	–											
2. Gender	1.24	0.43	1.00-2.00	–	−0.19**	–										
3. Hierarchical level	2.00	0.98	1.00-5.00	–	0.12**	–0.01	–									
4. Tenure	12.42	8.64	0.00-45.00	–	0.72**	−0.20**	0.04	–								
5. Honesty-Humility	3.92	0.47	1.90-5.00	0.08	0.06**	0.07**	–0.02	0.05*	(0.62)							
6. Openness to Exp^a^	3.63	0.51	1.30-5.00	0.06	−0.06**	0.02	0.01	−0.08**	0.08**	(0.72)						
7. Self-transcendence	0.00	1.00	−2.33-2.48	0.14	0.12**	–0.03	–0.03	0.14**	0.34**	0.17**	(0.61)					
8. Openness to change	0.00	1.00	−2.12-2.55	0.10	−0.08**	0.03	0.07**	−0.10**	0.06**	0.06**	0.08**	(0.75)				
9. Perceived UM^b^	2.69	0.54	1.00-4.58	0.03	–0.01	–0.03	0.04	–0.01	−0.43**	0.00	−0.19**	–0.04	(0.80)			
10. Risk aversion	2.37	0.58	1.00-5.00	0.09	0.16**	0.07**	–0.03	0.15**	−0.06**	−0.24**	−0.08**	−0.29**	0.05*	(0.74)		
11. Rel comp level^c^	3.99	0.81	2.00-6.00	0.06	−0.10**	−0.11**	–0.02	−0.06**	−0.17**	0.08**	−0.10**	–0.01	0.18**	−0.12**	–	
12. Compensation var^d^	34.26	15.10	0-100.00	0.15	−0.08**	−0.09**	–0.00	–0.03	0.03	0.12**	0.11**	–0.02	–0.01	−0.17**	0.08**	–

*N = 2210. Numbers in parentheses along the diagonal indicate alpha reliabilities, with the exception of Self-Transcendence and Openness to Change. For these two variables (represented using component scores), theta reliabilities are presented. Reliability is not shown for preference for relative compensation level as this scale contained two items only, or for preference for compensation variability as well as control variables, as these were assessed using one item only.*

*^*a*^Openness to Exp = openness to experience from the HEXACO model (HEXACO-PI-R: Ashton and Lee, 2009).*

*^*b*^Perceived UM = perceived utility of money.*

*^*c*^Rel comp level = preference for higher relative compensation level.*

^*d*^Compensation var = preference for compensation variability.

*p < 0.05.

**p < 0.01.

#### Personality

Honesty-Humility and Openness to Experience were measured using 20 items (10 items per factor scale) of the 60-item HEXACO Personality Inventory (HEXACO-60; Ashton and Lee, 2009).

#### Values

The Work Values Survey (WVS; Cable and Edwards, 2004) was used to measure both value axes. Although the original WVS contains 24 statements belonging to eight work value dimensions covering all of Schwartz’s universal values (Cable and Edwards, 2004), some adaptations were made to fit the current study. This included separating the achievement and hedonism scales and revising the items to still reflect the underlying values but to include non-pay-related content so as to prevent predictor–mediator content overlap. The final scale contained 27 items, which can be found in Supplementary Table B1. For each item, the participants responded to the question “How important is this to you in your work?” on a scale ranging from 1 (not important at all) to 5 (extremely important).

For the purpose of this study, we focused on the two key bipolar value axes, self-transcendence *versus* self-enhancement as well as openness to change *versus* conservation, herein referred to as self-transcendence and openness to change, respectively. The construction process of the two scales is described in Supplementary Appendix C.^4^ Ultimately, the component scores for the two separate dimensions were retained and used for further analysis.

#### Perceived Utility of Money

Perceived utility of money was assessed using a subset of items from Tang’s (1993) money ethics scale. The items included were those capturing the perceived level of achievement, respect, and freedom an individual derives from money. The items evaluating good, evil, and budget-related connotations associated with money were excluded. The adapted scale is presented in Supplementary Table D1.

#### Work-Related Risk Aversion

Work-related risk aversion was measured using six items adapted from the risk aversion scale created by Cable and Judge (1994).

#### Preferred Compensation Variability

To assess preference for compensation variability, the individuals were asked to divide the total monetary value of a hypothetical compensation package upon achieving budgeted performance (100%). The participants were asked to divide the total value into base salary, annual incentive, and long-term incentive [see Supplementary Table E1]. Preference for variability was defined as the proportion of variable elements (% allocated to annual incentive and long-term incentive) in the total package (100%).

#### Preferred Relative Compensation Level

Preference for higher relative compensation level was measured using two items developed for this study, assessing the individuals’ desire to earn more/less than others conducting a similar work. The items are presented in Supplementary Table F1. The responses were indicated on a six-point scale, ranging from 1 (lower) to 6 (higher). The items were significantly correlated (*r* = 0.52, *p* < 0.001).

### Data Structure

As the participants were nested within 12 subsidiary companies, it was necessary to examine the partition of focal variable variance into within- and between-levels of operating company. Intraclass coefficients (ICC1) ranged from .03 (perceived utility of money) to .15 (preference for compensation variability), suggesting that between 3 and 15% of the variance resided at the level of the operating company (Meyers et al., 2006; see Table 1 for ICC1 values), that is, the operating company accounted for a non-trivial amount of variance in some, but not in all, study variables. Given this range of ICC values, it would be infeasible to meaningfully account for a hierarchical structure across all variables (Kozlowski, 2012). As both the majority of variance and theoretical interest in the present study reside at the individual (within) level, we removed the little existing variance at the level of operating company (*i*.*e*., at the between-level) by group mean centering all observed variables (Enders and Tofighi, 2007). Relations were subsequently estimated at the level of the individual.

## Results

Descriptive statistics, intraclass coefficients (ICC1), and zero-order correlations based on the raw (non-centered) data are presented in Table 1.

### Hypotheses 1–4

Zero-order correlations were calculated using both the raw (presented in Table 1) and group-mean-centered data^5^, and hypothesized predictor–criterion relations were evaluated using both sets of correlations. Generally, hypotheses 1, 2, and 3 received support, using both raw and group-mean-centered data. Specifically, Honesty-Humility exhibited a small- to medium-sized^6^ significant negative relation with preference for higher relative compensation level when using both raw (*r* = −0.17, *p* < 0.001) and centered data (*r* = −0.20, *p* < 0.001). Openness to Experience also demonstrated a small yet significant positive relation with compensation variability when using the raw (*r* = 0.12, *p* < 0.001) and centered data (*r* = 0.05, *p* = 0.011). Lastly, the results also revealed a small significant positive relation between self-transcendence and preference for higher relative compensation level when using the raw (*r* = −0.10, *p* < 0.001) and centered data (*r* = −0.16, *p* < 0.001). However, whereas openness to change demonstrated a small yet significant positive relation with preference for compensation variability when using the centered data (*r* = 0.06, *p* = 0.004), this was not the case when using raw data (*r* = −0.02, *p* = 0.387). Therefore, hypothesis 4 was supported only when using the group-mean-centered data.

### Mediated Relations

**FIGURE 1:**
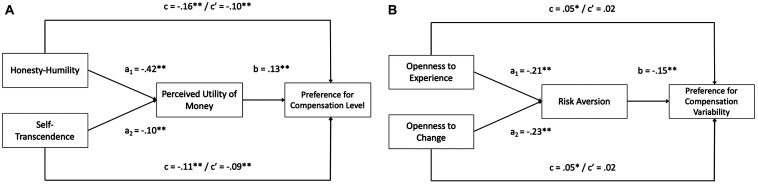
**(A)** Standardized path coefficients for relations proposed in hypothesis 5. **(B)** Standardized path coefficients for relations proposed in hypothesis 6. Standardized path estimates for hypothesized mediations. c = total effect; c’ = direct effect. Path coefficients for control variables are not included in the above figures, despite being present in the analysis. ^∗^*p* < 0.05, ^∗∗^*p* < 0.01.

To examine the plausibility of hypotheses 5 (a and b) and 6 (a and b), mediation analyses based on recommendations from Hayes (2018) were conducted^7^. The SPSS PROCESS macro v3.4 was used to evaluate direct and indirect effects in the proposed models through bias-corrected bootstrapping, with 5,000 resamples. To control for the effects of demographic factors, the following variables were also listed as covariates in all models: age, gender, hierarchical level^8^, and tenure within the organization.^9^ The path estimates for the mediation model outlined in hypothesis 5 can be found in Table 2 and Figure 1A. The results demonstrated a significant indirect effect of Honesty-Humility to preference for higher relative compensation level through perceived utility of money [β = −0.05, Boot SE = 0.01, 95% CI (−0.07, −0.03)]. In addition, self-transcendence was found to exert a significant indirect effect on preference for higher relative compensation level through perceived utility of money [β = −0.01, Boot SE = 0.00, 95% CI (−0.02, −0.01)]. Consequently, the results provide support for hypotheses 5a and 5b. As can be seen in Figure 1A, after accounting for the indirect effects through perceived utility of money, significant direct effects between each predictor and preference for higher relative compensation level remained. Included in the mediation model but not the subject of the research hypotheses were control variables of age, gender, hierarchical level, and tenure. Gender^10^ was found to be a significant predictor of preference for higher relative compensation level, both with and without the mediator in the model (see Table 2).

**TABLE 2:** Path coefficients for relations proposed in hypothesis 5.

	DV: Perceived utility of money					DV: Preference for higher relative compensation level				
		
				Lower	Upper				Lower	Upper
	β	B	SE	95% CI	95% CI	β	B	SE	95% CI	95% CI
**Control variables**										
Age	0.04	0.00	0.00	–0.00	0.01	–0.05	–0.00	0.00	–0.01	0.00
Gender	0.02	0.02	0.03	–0.03	0.07	–0.08	−0.15**	0.04	–0.23	–0.08
Hierarchical level	0.04	0.02*	0.01	0.00	0.04	0.00	0.00	0.02	–0.03	0.04
Tenure	–0.01	–0.00	0.00	–0.00	0.00	–0.03	–0.00	0.00	–0.01	0.00
**IVs**										
Honesty-Humility	–0.42	−0.50**	0.02	–0.55	–0.45	–0.16	−0.27**	0.04	–0.35	–0.20
Self-transcendence	–0.10	−0.06**	0.01	–0.08	–0.03	–0.11	−0.09**	0.02	–0.13	–0.05
**Mediator**										
Perceived utility of money	–	–	–	–	–	–	–	–	–	–

	**DV: Preference for higher relative compensation level**									
	
				**Lower**	**Upper**					
	**β**	**B**	**SE**	**95% CI**	**95% CI**					

**Control variables**										
Age	–0.05	–0.01	0.00	–0.01	0.00					
Gender	–0.08	−0.16**	0.04	–0.24	–0.08					
Hierarchical level	–0.00	–0.00	0.02	–0.04	0.03					
Tenure	–0.03	–0.00	0.00	–0.01	0.00					
**IVs**										
Honesty-Humility	–0.10	−0.18**	0.04	–0.26	–0.10					
Self-transcendence	–0.09	−0.08**	0.02	–0.12	–0.04					
**Mediator**										
Perceived utility of money	0.13	0.19**	0.03	0.12	0.25					

N = 2210. β = standardized path estimates. B = unstandardized path estimates. *p < 0.05. ** *p* < 0.01.

The path estimates for the mediation model outlined in hypothesis 6 can be found in Table 3 and Figure 1B. The results revealed a significant indirect effect from Openness to Experience to preference for compensation variability through risk aversion [β = 0.03, Boot SE = 0.01, 95% CI (0.02, 0.04)]. Additionally, openness to change also exhibited a significant indirect effect on preference for compensation variability through risk aversion [β = 0.03, Boot SE = 0.01, 95% CI (0.02, 0.05)]. As a result, the findings also provide support for hypotheses 6a and 6b. After accounting for the indirect effects through risk aversion, no significant direct effects between each predictor and preference for compensation variability remained (see Figure 1B). Moreover, also in the mediation model, but again not the focus of the research hypotheses, were all four control variables. Age, gender, and hierarchical level were all found to be significant predictors of preference for compensation variability when the mediator was not present in the model (see Table 3), although only gender and hierarchical level remained significant when the mediator was included in the model.

**TABLE 3:** Path coefficients for relations proposed in hypothesis 6.

	DV: Risk aversion					DV: Preference for compensation variability				
		
	β	B	SE	Lower 95% CI	Upper 95% CI	β	B	SE	Lower 95% CI	Upper 95% CI
**Control variables**										
Age	0.08	0.01*	0.00	0.00	0.01	–0.06	−0.11*	0.05	–0.21	–0.01
Gender	0.10	0.13**	0.03	0.08	0.19	–0.06	−1.97**	0.72	–3.39	–0.55
Hierarchical level	–0.06	−0.03*	0.01	–0.06	–0.01	0.06	0.88**	0.33	0.25	1.52
Tenure	0.08	0.01	0.00	0.00	0.01	0.02	0.03	0.05	–0.07	0.13
**IVs**										
Openness to Experience	–0.21	−0.23**	0.02	–0.28	–0.19	0.05	1.30*	0.61	0.12	2.49
Openness to change	–0.23	−0.13**	0.01	–0.16	–0.11	0.05	0.73*	0.32	0.11	1.35
**Mediator**										
Risk aversion	–	–	–	–	–	–	–	–	–	–

	**DV: Preference for compensation variability**									
	
	**β**	**B**	**SE**	**Lower 95% CI**	**Upper 95% CI**					

**Control variables**										
Age	–0.05	–0.09	0.05	–0.19	0.01					
Gender	–0.04	−1.47*	0.72	–2.88	–0.05					
Hierarchical level	0.05	0.76*	0.32	0.12	1.39					
Tenure	0.03	0.05	0.05	–0.05	0.15					
**IVs**										
Openness to Experience	0.02	0.44	0.61	–0.77	1.64					
Openness to change	0.02	0.23	0.32	–0.40	0.86					
**Mediator**										
Risk aversion	–0.15	−3.73**	0.57	–4.85	–2.61					

N = 2210. β = standardized path estimates. B = unstandardized path estimates. *p < 0.05. **p < 0.01.

## Discussion

The present study examined dispositional predictors of employee preferences for two key aspects of compensation. As anticipated, Honesty-Humility exhibited a small to medium, statistically significant negative relation with preference for higher relative compensation level, which was partially mediated by perceived utility of money. That is, the relation between Honesty-Humility and preference for higher relative compensation level can be at least partly explained by the fact that individuals who score low in Honesty-Humility also tend to be characterized by a strong inclination to prioritize money (Ashton et al., 2010; Lee et al., 2013). However, this result also suggests that a portion of the relation between Honesty-Humility and preference for higher relative compensation level can be attributed to non-financial considerations. One such example could be a more general belief that low scorers simply feel entitled to more than others (Lee and Ashton, 2005). Similarly, self-transcendence also exhibited a small yet statistically significant negative relation with preference for higher relative compensation level, which was partially mediated by perceived utility of money. This partial mediation suggests that the relation between self-transcendence and preference for higher relative compensation level is only partly attributable to the fact that financial gains can satiate one’s desire for personal gain and power (Schwartz, 1994; Winter, 1973). This indicates that other explanations, such as a more general prioritization of the self over others (*i*.*e*., opposite to the universalism value represented; Schwartz, 1994), may also be at play when examining the relation between this value dimension and preference for higher relative compensation level.

Openness to Experience demonstrated a small yet statistically significant positive relation with preference for compensation variability, replicating this finding from previous research (Vandenberghe et al., 2008). Furthermore, this positive relation was fully mediated by individuals’ level of risk aversion. This finding suggests that the relation between this trait and preference for compensation variability can be explained by the fact that individuals who are high in Openness to Experience are more likely to take risks compared to others (De Vries et al., 2009). In turn, previous research has linked risk attitudes to preferences for compensation variability, as highly variable compensation schemes open employees up to some level of risk (Cable and Judge, 1994). Similarly, the value axis of openness to change demonstrated a small yet statistically significant positive relation with preference for compensation variability^11^, and this relation was fully mediated by individuals’ level of risk aversion. Similar to Openness to Experience, this full mediation is likely attributable to the fact that individuals who are open to change and new experiences are often more inclined to take risks (De Vries et al., 2009), which again, in turn, has been previously found to relate to compensation variability preferences (Cable and Judge, 1994).

Whereas not the focus of hypothesized relations, the results also suggest that men are more likely to prefer higher levels of, and more variable, compensation compared to women. This is not completely unsurprising for compensation level, as preferences may be influenced by expectations founded on decades of pay inequality between genders. Additionally, the results demonstrate that older individuals are less likely to prefer more variable compensation, possibly due to the fact that individuals become more risk-averse with age (Pålsson, 1996). This interpretation is also supported by the fact that age became non-significant as a predictor once risk aversion was added into the model. Lastly, individuals at higher levels of the organization appear to be more likely to prefer more variable compensation compared to those at lower levels. This may be due to heightened perceptions of autonomy over one’s work or increased self-efficacy due to promotions to higher levels of the organization, both of which could plausibly attenuate perceived risk associated with variable compensation.

### Implications

Earlier studies investigating antecedents to compensation preferences have primarily emphasized the role of applicant/employee performance level (Cadsby et al., 2007; Shaw and Gupta, 2007) and, to a lesser extent, characteristics such as cultural orientation (Yeganeh and Su, 2011) and personality traits (Cable and Judge, 1994; Vandenberghe et al., 2008; Westerman et al., 2009). Findings from the current study offer a more nuanced understanding of how dispositional traits (*i*.*e*., HEXACO personality traits and values) predict employee preferences for two key aspects of corporate compensation.

In addition, whereas the observed relations examined were generally small to medium in size, it is important to note that even small effects can accumulate across individuals and over time to produce a substantial impact (Funder and Ozer, 2019). Findings from the present study pose several practical implications for organizations, that is, previous research has demonstrated that certain characteristics and preferences may render individuals more likely to be drawn to select features of an organization’s compensation structure and therefore make them more likely to be attracted to, selected into, and retained within a given organization on the basis of such preferences [sorting effects; see Gerhart et al. (2009) for a review]. Therefore, aspects of an organization’s compensation system may ultimately influence important qualities of its workforce. Consequently, gaining insight into the relations among individual characteristics and preferences for various forms of compensation is of utmost importance for organizations to better understand the implications of decisions around compensation system features on workforce composition and its career aspirations.

### Limitations, Future Directions, and Conclusion

The present study is subject to select limitations worth noting. Data were derived from single-source self-report surveys, rendering the study’s findings vulnerable to common method bias. Whereas many variables under examination pertain to information that would not have been outwardly observable by sources other than the participants themselves, such as values and preferences, for others, such as personality traits, it may have been useful to collect data from additional sources. It should also be noted, however, that there has been relatively little evidence of method or source factors in the HEXACO-PI-R (*e*.*g*., Ashton and Lee, 2010). Regardless, future research may choose to replicate the findings from the present study, incorporating various sources of data to rule out any possible concerns regarding common method bias.

It is also important to note that Honesty-Humility and Openness to Experience may not be the only personality traits relevant to the prediction of compensation preferences. These traits were selected based on their particular relevance to the criteria examined in this study and because they exhibit some conceptual similarity with the value axes from Schwartz (1994) circumplex (Lee et al., 2009). As the study involved a field-based sample, there were explicit survey length limitations set by management, and therefore all six HEXACO personality traits could not be included. Future research should explore the prediction of compensation preferences from all six HEXACO personality traits.

It is also important to address the fact that the internal consistency reliabilities for a subset of the measures used in this study (*e*.*g*., Honesty-Humility) could be considered as below desirable levels. Future research may seek to re-examine the relations from this study using a longer measure of HEXACO personality (*e*.*g*., HEXACO-100; Lee and Ashton, 2018), which is likely to produce higher alpha reliability coefficients for factor scales. Additionally, the component structure of the WVS (Cable and Edwards, 2004) found in the present study did not adequately support a circumplex structure. We circumvented this problem by retaining factor-analytically derived component scores of typical markers of openness to change and self-transcendence (see Supplementary Appendix C). Future research may like to further investigate the structural properties of the WVS and/or draw upon an alternative measure to re-examine the proposed relations involving personal values.

Moreover, it is important to note that the sample was obtained in only one company, potentially causing restriction of range effects. However, it should be noted that the study was conducted in an originally highly decentralized global company, in which the subsidiaries were not started as greenfield operations but were obtained through takeovers. Importantly, possible restriction of variance effects may have resulted in our findings being conservative estimates of true effect sizes in the population. Lastly, a final limitation to the current study is that the employees’ actual compensation level was not measured and therefore not included in analyses despite the fact that current compensation may be a predictor of preferred level of compensation (cf. Rice et al., 1990). Future studies examining predictors of preference for compensation level may consider controlling for this variable.

In summary, the present study offers evidence in support of personality traits and values as antecedents to employee preferences for compensation variability and level. Moreover, the current study furthers our understanding of these relations by demonstrating support for two additional traits (*i*.*e*., risk aversion and perceived utility of money) as mediating mechanisms. By gaining an understanding of the dispositions characteristic of individuals likely to prefer certain compensation system dimensions (*i*.*e*., relative compensation level and compensation variability), organizations can better tailor their compensation systems to attract and retain individuals deemed as desirable or to deter those who are not.

## Data Availability Statement

The raw data supporting the conclusions of this article will be made available by the authors, without undue reservation.

## Ethics Statement

The studies involving human participants were reviewed and approved by The Scientific and Ethical Review Board (VCWE) of the Faculty of Behavior and Movement Sciences, VU University Amsterdam. Written informed consent for participation was not required for this study in accordance with the national legislation and the institutional requirements.

## Author Contributions

AJ performed conceptualization, formal analysis, writing original draft, and review and editing. OW performed conceptualization, methodology, and investigation. RdV performed writing review and editing and supervision. All authors contributed to the article and approved the submitted version.

## Conflict of Interest

The Vrije Universiteit Amsterdam obtains royalties for the commercial use of the HEXACO personality inventory, which are used by the last author for research purposes. OW was employed by the company Heineken International B.V., Amsterdam, at the time of the research. The remaining authors declare that the research was conducted in the absence of any commercial or financial relationships that could be construed as a potential conflict of interest.

## Footnotes

1 The ordering of the axis poles has been reversed to remain conceptually consistent with HEXACO Honesty-Humility.

2 At the time the study was conducted, it was not necessary to obtain institutional ethical approval. However, the study was conducted in full compliance with organizational and institutional ethical norms, and thus *post hoc* ethical approval (VCWE-2020-040) was obtained.

3 A total of 2,221 provided complete questionnaires; however, 11 individuals incorrectly labeled the operating company at which they were located and were therefore removed from analyses.

4 The decision was made to focus on four core values subsumed under the four ends of the axes, which were chosen based on both empirical (*i*.*e*., high reliabilities and expected zero-order correlations) and theoretical reasoning (*i*.e., representative). Stimulation and security were retained to represent the axis of openness to change (*versus* conservation), referred to as openness to change. Additionally, values of universalism and power were retained to represent the axis of self-transcendence (*versus* self-enhancement), referred to as self-transcendence.

5 This data is available upon request from the first author of the paper.

6 Based on the effect size guidelines for individual difference research provided by Gignac and Szodorai (2016).

7 Using group-mean-centered data.

8 Hierarchical level here was measured using a rating scale ranging from 1 to 5, where a response of 5 indicated that a participant was the managing director of the operating company (and therefore at the highest level of management at the local operating company) and 1 indicated that they were more than three hierarchical levels below the managing director.

9 When a mediation model contains more than a single independent variable, Hayes (2018) suggests that PROCESS be run *k* times (where *k* = the number of independent variables). For each run, one independent variable is to be listed as such, with the remaining independent variables to be listed as covariates. By default, covariates are treated as independent variables, and each run estimates the effects for the variable listed as the independent variable. This procedure was adopted in the present study.

10 Where men were coded as 1 and women were coded as 2.

11 This is again only the case for mean-centered data. When using raw data, there does not appear to be a significant direct effect from openness to change to preference for compensation variability.
