# Supplementary material for: Compensation Preferences: The Role of Personality and Values
Source: Front Psychol. 2021 Apr 7;12:550919. doi: 10.3389/fpsyg.2021.550919 (PMC8058456; doi:10.3389/fpsyg.2021.550919)
Supplement: Supplementary file 1 [file Data_Sheet_1.docx]

**APPENDIX A**

**Table A1**

*Demographic Information Broken Down by Individual Operating Company (Country)*

| Company (country) | N | % Female | Mean age (SD) | Mean tenure (SD) |
| --- | --- | --- | --- | --- |
| Greece | 127 | 6.3 | 46.51 (7.39) | 17.94 (8.72) |
| Mexico | 274 | 11.7 | 40.09 (7.84) | 13.12 (8.58) |
| Poland | 300 | 23.3 | 42.83 (8.28) | 14.56 (8.00) |
| Brazil | 93 | 30.1 | 36.60 (7.57) | 8.27 (6.38) |
| France | 156 | 40.4 | 39.45 (7.92) | 10.12 (8.40) |
| Italy | 99 | 17.2 | 46.02 (7.04) | 13.89 (7.24) |
| Netherlands | 218 | 21.6 | 41.75 (8.24) | 12.70 (9.61) |
| Romania | 156 | 37.2 | 37.24 (7.26) | 7.87 (5.94) |
| Russia | 104 | 49.0 | 36.29 (8.17) | 5.54 (3.06) |
| Spain | 219 | 23.7 | 43.22 (7.72) | 16.06 (9.11) |
| UK | 290 | 27.2 | 41.30 (8.59) | 11.02 (8.89) |
| Nigeria | 174 | 10.9 | 39.94 (7.02) | 12.62 (7.62) |

*Note*. Participants occupied roles from a wide range of functions specific role types included commerce (34.8%), corporate relations (1.1%), finance (12.0%), general management (3.1%), human resources (6.9%), IT (4.5%), legal (1.4%), supply chain (25.5%), and other (10.6%).

**APPENDIX B**

**Table B1**

*Dimensions and Items from the Adjusted Work Values Survey (WVS; Cable & Edwards, 2004) Mapped onto Schwartz’s Universal Values*

| Schwartz’s conceptual dimensions | Schwartz’s universal values | Specific items used in the present study |
| --- | --- | --- |
| Self-transcendence | Universalism | Making the world a better place |
|  |  | Being of service to society |
|  |  | Contributing to humanity |
|  | Benevolence | Forming relationships with co-workers |
|  |  | Getting to know your fellow workers quite well |
|  |  | Developing close ties with co-workers |
| Self-enhancement | Achievement | Being the best at what I do (adjusted) |
|  |  | Outperforming colleagues (adjusted) |
|  |  | Performing to the best of my ability (adjusted) |
|  | Hedonism | Having an enjoyable job (adjusted) |
|  |  | Enjoying the advantages of my job (adjusted) |
|  |  | Having a good time at work (adjusted) |
|  | Power | Gaining respect |
|  |  | Obtaining status |
|  |  | Being looked up to by others |
| Conservation | Security | Being certain of keeping my job |
|  |  | Being sure I will always have a job |
|  |  | Being certain my job will last |
|  | Conformity/tradition | Distinct reporting relationships |
|  |  | A clear chain of command |
|  |  | Definite lines of authority |
| Openness to change | Stimulation | Doing a variety of things |
|  |  | Doing something different every day |
|  |  | Doing many different things on the job |
|  | Self-direction | Doing my work in my own way |
|  |  | Determining the way my work is done |
|  |  | Making my own decisions |

*Note.* Respondents were asked to answer “How important is this to you in your work?”. Responses ranged from 1 (*Not important at all*) to 5 (*Extremely important*). The Achievement and Hedonism scales that were originally combined in the WVS were separated for the present study.

**APPENDIX C**

**Structure of Values Dimensions**

To examine whether the structure of the WVS in the present study aligned with the Schwartz’ (1992) circumplex model of human values, the items were examined using exploratory factor analysis.^[[1]](#footnote-1)^ Firstly, all 27 items were submitted to an initial principal component analysis (PCA) with an oblique rotation (direct oblimin) to determine feasibility of analysis and to obtain eigenvalues for each factor in the data. The Kaiser-Meyer-Olkin value of .84 indicated sampling adequacy, and Bartlett’s test of sphericity was significant, *χ^2^*(351) = 20, 444.30, *p* < .001. Eight components had eigenvalues over Kaiser’s criterion of 1, and in combination explained 63.45 % of the variance. The scree plot was somewhat ambiguous, but the inflection point justified retaining a 7- or 8-component structure. A more sophisticated parallel analysis (O’Connor, 2000) based on 9000 data sets confirmed an 8-component structure. The item loadings for the 8-components after rotation were highly representative of the original values in the WVS (see Table C2 for component loadings), however this structure was not well aligned with the values of the original circumplex model (Schwartz, 1992). The component structure in the present study suggests that the values are fairly independent of one another as opposed to belonging to bipolar axes. In an attempt to circumvent measurement issues due to the component structure of the scale, the decision was made to focus on four core values subsumed under the four ends of the axes (i.e., one for each type), which were chosen based on both empirical (i.e., high reliabilities, expected zero-order correlations) and theoretical reasoning (i.e. representative). Consequently, values of Stimulation and Security were retained to represent the axis of Openness to Change (versus Conservation), referred to as Openness to Change. Additionally, values of Universalism and Power were retained to represent the axis of Self-Transcendence (versus Self-Enhancement), referred to as Self-Transcendence. The items belonging to the four values outlined above were then ipsatized. In the ipsatization process, 27 participants demonstrated a standard deviation of zero across included items. As these individuals did not demonstrate patterns consistent with careless responding, individuals were assigned a score of zero across all ipsatized items, as opposed to removing them from the analyses. To obtain scales that were highly representative of the two axes, items were split in accordance with the two axes to which they belonged, and each set of items was submitted to a PCA separately, extracting a one-component structure. Component loadings ranged from -.625 to .729 for Openness to Change items and -.525 to .726 for Self-Transcendence items (see Table C3 and C4 for all component loadings). The component scores were saved and subsequently used in data analyses as opposed to the original scale scores.

**Table C1**

*Bivariate Correlations Among Values Scales of the WVS*

|  | Universalism | Benevolence | Achievement | Hedonism | Power | Security | Conformity /tradition | Stimulation | Self-direction |
| --- | --- | --- | --- | --- | --- | --- | --- | --- | --- |
| Universalism |  |  |  |  |  |  |  |  |  |
| Benevolence | .34 |  |  |  |  |  |  |  |  |
| Achievement | .24 | .29 |  |  |  |  |  |  |  |
| Hedonism | .21 | .31 | .29 |  |  |  |  |  |  |
| Power | .27 | .33 | .51 | .35 |  |  |  |  |  |
| Security | .27 | .32 | .32 | .31 | .46 |  |  |  |  |
| Conformity/tradition | .32 | .28 | .29 | .24 | .35 | .45 |  |  |  |
| Stimulation | .14 | .16 | .21 | .28 | .18 | .08 | .14 |  |  |
| Self-direction | .04 | .16 | .25 | .29 | .21 | .09 | .10 | .37 |  |

*N* = 2210.

**Table C2**

*Component Loadings from the Pattern Matrix for Adapted WVS Items Used in the Present Study*

| Items | Component | | | | | | | |
| --- | --- | --- | --- | --- | --- | --- | --- | --- |
|  | 1 | 2 | 3 | 4 | 5 | 6 | 7 | 8 |
| Being sure I will always have a job (Security) (14) | **.86** | .01 | .00 | .08 | -.01 | .03 | .02 | -.01 |
| Being certain of keeping my job (Security) (6) | **.84** | .02 | -.01 | .07 | -.02 | .12 | -.02 | -.10 |
| Being certain my job will last  (Security) (22) | **.82** | .03 | .06 | .11 | -.03 | .01 | -.00 | -.03 |
| Enjoying the advantages of my job (Hedonism) (20) | .30 | .04 | .08 | -.07 | -.23 | .03 | .26 | .13 |
| Doing many different things on the job (Stimulation) (24) | .08 | **.85** | -.07 | -.05 | -.01 | -.02 | -.06 | .06 |
| Doing a variety of things  (Stimulation) (8) | -.01 | **.84** | -.06 | .01 | -.05 | -.05 | .02 | -.05 |
| Doing something different every day (Stimulation) (16) | -.03 | **.73** | .15 | .03 | .10 | .05 | .01 | .04 |
| Making the world a better place (Universalism) (1) | -.02 | -.02 | **.89** | -.00 | .05 | .01 | .01 | -.10 |
| Contributing to humanity  (Universalism) (18) | .00 | -.01 | **.88** | .01 | -.02 | .01 | .04 | .04 |
| Being of service to society  (Universalism) (10) | .06 | .05 | **.74** | .01 | -.16 | -.03 | -.01 | -.00 |
| Defined lines of authority (Conformity/tradition) (23) | .08 | .03 | -.04 | **.85** | -.02 | -.08 | .01 | .06 |
| A clear chain of command (Conformity/tradition) (15) | .08 | .01 | .03 | **.85** | .04 | -.02 | .02 | .00 |
| Distinct reporting relationships (Conformity/tradition) (7) | .01 | -.02 | .02 | **.72** | -.06 | .04 | .04 | -.04 |
| Getting to know your co-workers quite well (Benevolence) (11) | .03 | .01 | .05 | .03 | **-.84** | -.12 | -.03 | .07 |
| Developing close ties with co-workers (Benevolence) (19) | .04 | .01 | .16 | -.02 | **-.78** | -.08 | -.05 | .10 |
| Forming relationships with co-workers (Benevolence) (3) | -.02 | -.00 | -.07 | .05 | **-.76** | .18 | -.01 | -.12 |
| Having an enjoyable job  (Hedonism) (4) | .06 | .07 | .01 | .03 | .04 | **.81** | -.05 | .00 |
| Having a good time at work  (Hedonism) (27) | .09 | -.03 | .04 | -.08 | .05 | **.73** | -.08 | .22 |
| Gaining respect  (Power) (5) | .05 | .03 | -.07 | .09 | -.22 | **.48** | .26 | -.13 |
| Outperforming colleagues  (Achievement) (12) | .03 | -.01 | -.04 | .06 | .00 | -.17 | **.77** | .14 |
| Being the best at what I do  (Achievement) (2) | -.13 | .08 | .09 | .08 | .00 | .13 | **.70** | -.16 |
| Obtaining status  (Power) (13) | .26 | -.02 | -.01 | .04 | -.01 | -.10 | **.62** | .09 |
| Being looked up to by others  (Power) (21) | .26 | -.02 | .13 | -.01 | .06 | .01 | **.50** | .09 |
| Performing to the best of my ability  (Achievement) (26) | -.12 | .06 | .06 | .04 | -.12 | .26 | **.40** | .07 |
| Doing my work in my own way  (Self-direction) (9) | .06 | .06 | -.09 | -.15 | -.03 | .04 | .06 | **.72** |
| Making my own decisions  (Self-direction) (25) | -.09 | .09 | -.08 | .02 | -.06 | .02 | .18 | **.66** |
| Determining the way my work is done (Self-direction) (17) | -.09 | .04 | .11 | .27 | .02 | .14 | -.14 | .**61** |

*Note.* *N* = 2210. Component loadings greater than .40 are shown in bold. Numbers in parentheses after the items indicate the order in which the items were presented to participants.

**Table C3**

*Component Loadings from Component Matrix for Openness to Change (vs. Conservation)*

| Items | Component loadings |
| --- | --- |
| 1. Being certain of keeping my job (Security) | .683 |
| 2. Being sure I will always have a job (Security) | .729 |
| 3. Being certain that my job will last (Security) | .716 |
| 4. Doing a variety of things (Stimulation) | -.609 |
| 5. Doing something different every day (Stimulation) | -.612 |
| 6. Doing many different things on the job (Stimulation) | -.625 |

*Note.* For analyses, the component scores were multiplied by (-1), in order to have the scores align with the direction of the theoretical construct of HEXACO Openness to Change. *N* = 2210.

**Table C4**

*Component Loadings from Component Matrix for Self-Transcendence (vs. Self-Enhancement)*

| Items | Component loadings |
| --- | --- |
| 1. Making the world a better place (Universalism) | .705 |
| 2. Being of service to society (Universalism) | .629 |
| 3. Contributing to Humanity (Universalism) | .726 |
| 4. Gaining respect (Power) | -.321 |
| 5. Obtaining status (Power) | -.525 |
| 6. Being looked up to by others (Power) | -.489 |

*Note. N* = 2210.

**APPENDIX D**

**Table D1**

*Items for the Perceived Utility of Money Scale Adopted from the Money Ethics Scale (Tang, 1993)*

| Items |
| --- |
| 1. Money can buy everything. |
| 2. Money represents one’s achievement |
| 3. Money is the most important thing (goal) in my life. |
| 4. Money is a symbol of success. |
| 5. Money is honourable. |
| 6. Money can bring you many friends. |
| 7. Money helps you express your competence and abilities. |
| 8. Money makes people in the community respect you. |
| 9. Money in the bank is a sign of security. |
| 10. Money means power. |
| 11. Money gives you autonomy and freedom. |
| 12. Money can give you the opportunity to be what you want to be. |

*Note.* Responses ranged from 1 (*strongly disagree*) to 5 (*strongly agree*).

**APPENDIX E**

**Table E1**

*Measure of Preferred Compensation Variability*

| Package elements | Description |
| --- | --- |
| Base salary | A cash part of your compensation package (including allowances) with a fixed value. Base salary is generally paid to you in monthly instalments. |
| Annual incentive | A cash part of your compensation package with a variable value. It is paid to you at the targeted level if you achieve exactly the individual performance objectives that were set for you by your employer. If your individual performance exceeds the performance objectives, it can become up to twice as high as expected. If you fail to meet these objectives, there may be a lower payout or no pay out at all. |
| Long-term incentive | A part of your compensation package paid to you in company shares (stock). On an annual basis, you receive a fixed percentage of your base salary in share entitlements against the share price at that moment. These entitlements will become actual tradable shares after a three year performance period, provided you remain in company service. If the company achieves exactly its predefined performance objectives during this period, all entitlements will become actual shares. If the company performance exceeds these objectives, you may receive up to twice as many shares. If the company fails to meet the objectives, you may receive fewer shares or no shares at all. |

*Note.* Participants responded to the question: “If your company granted you the freedom to create your own compensation package from these three elements, what percentage mix would you choose?”

**APPENDIX F**

**Table F1**

*Items for Preferred Relative Compensation Level Developed For Use in This Study*

| Items |
| --- |
| 1. Compared to my co-workers, I prefer my total compensation package to be… |
| 2. Compared to people doing a comparable job (in terms of level and responsibilities) in other companies in my industry, I prefer my total compensation package to be… |

*Note.* Responses ranged from 1 (*lower*) to 6 (*higher*).

References

Cable, D. M., & Edwards, J. R. (2004). Complementary and supplementary fit: a theoretical and empirical integration. *Journal of Applied Psychology*, *89*(5), 822-834.

O'Connor, B. P. (2000). SPSS and SAS programs for determining the number of components using parallel analysis and Velicer's MAP test. [*Behavior Research Methods, Instrumentation, and Computers, 32*(3)*,*](https://www.springer.com/psychology/cognitive+psychology/journal/13428) 396-402.

Schwartz, S. H. (1992). Universals in the content and structure of values: Theoretical advances and empirical tests in 20 countries. *Advances in Experimental Social Psychology*, *25*(1), 1-65.

Tang, T. L. P. (1993). The meaning of money: Extension and exploration of the money ethic scale in a sample of university students in Taiwan. *Journal of Organizational Behavior*, *14*(1), 93-99.

1. Bivariate correlations among the values scales of the WVS are presented in Table C1. As can be seen from the table, all values exhibit positive relations with one another (to varying degrees). [↑](#footnote-ref-1)
